# Body weight modification experience among adolescents from Saudi Arabia

**DOI:** 10.3389/fpubh.2024.1323660

**Published:** 2024-04-19

**Authors:** Ibrahim M. Gosadi

**Affiliations:** Department of Family and Community Medicine, Faculty of Medicine, Jazan University, Jazan, Saudi Arabia

**Keywords:** adolescence, body weight, weight satisfaction, weight modification, Jazan, Saudi Arabia

## Abstract

**Background:**

Childhood malnutrition is a public health issue in developing countries, leading to a double burden of malnutrition, which is associated with both overweight and underweight.

**Objective:**

To assess body weight satisfaction and perception as well as body weight modification experiences, among adolescents from Saudi Arabia.

**Method:**

This study utilized a cross-sectional design targeting adolescents who attempted to modify their body weight. A questionnaire was constructed to measure their demographics, body weight satisfaction and perception, and experience concerning their weight modification attempts. The chi-square test was used to assess the association between the ability to modify weight and maintain the modification according to the ability to set an appropriate weight target based on the age and height of the adolescents, method of weight modification, and receipt of support to modify weight.

**Results:**

A total of 285 adolescents were recruited. More than half of the sample were female (58%); most respondents were secondary school students (73%). Nearly 45% had abnormal body weight where 12.6% were underweight, and 32.3% were overweight or obese. Most of the recruited sample were unsatisfied with their body weight (63%). Although 52% of the adolescents had a normal BMI, only 35% perceived their body weight as normal. Nearly 75% of the sample were able to modify their body weight. However, a smaller proportion were able to maintain the modification they achieved. The most frequently selected body weight modification method was dieting (83%), followed by exercise (69%). Only 40 adolescents (14%) reported consulting a physician regarding their body weight modification attempts. The most frequently reported source of support for weight modification was the family (51%), while the lowest frequency of support was reported concerning schools’ contribution (29%). Upon assessing factors associated with the ability to modify weight or maintain the modification, a higher frequency of adolescents who indicated they employed dieting behavior were able to maintain the modification compared to other weight modification practices (*p* < 0.05).

**Conclusion:**

The findings highlight the importance of collaboration between families, schools, and healthcare services to improve adolescent body image and ensure the adoption of healthy body weight modification practices among adolescents.

## Introduction

Abnormal body weight is considered a risk factor for multiple chronic non-communicable conditions and can increase the risk of death among vulnerable groups. According to the World Health Organization, in 2022, 390 million child and adolescents worldwide are considered overweight or obese. Additionally, in 2022, 149 million children were estimated to be underweight, and around 45% of deaths under the age of five are linked to undernutrition (1). Additionally, obesity during childhood and adolescence has been reported to increase the risk of development of chronic conditions during adulthood such as type 2 diabetes, hypertension, obstructive sleep apnea, and dyslipidemia (2, 3). The prevalence of underweight, overweight, and obesity varies according to countries and several socioeconomic factors.

Saudi Arabia is a developing, high-income country with a rising prevalence of overweight, obesity, and associated chronic non-communicable diseases. According to the World Health Survey report of Saudi Arabia, only 38% of Saudi adults have a normal body mass index (BMI), while 38% are considered overweight, 20% are considered obese, and nearly 2% are deemed underweight (4).

The increased prevalence of heightened BMI in the country can be partially explained by the obesogenic environment, augmented by a Westernized lifestyle and low levels of physical activity (5). However, the distribution of BMI abnormalities among Saudis can differ according to gender and age groups.

The current evidence concerning BMI profiling among Saudi children and adolescents nationwide is limited. Nonetheless, a recent systematic review that covered 18 studies with a total sample of 97,666 adolescents from Saudi Arabia indicates that the prevalence of overweight and obesity among them varies between 22 and 48% according to the year of study, age groups, and the region where the study was conducted (6). The prevalence of overweight and obesity among Saudi adolescents was higher among adolescents than younger children and has increased in recent years (7). A further increase in obesity among Saudi children is predicted for the future (7–9). Furthermore, a study that looked at the regional variation of overweight and obesity among adolescents in Saudi Arabia reported that southwestern regions of the country have a lower prevalence of overweight and obesity compared to other regions, suggesting a higher prevalence of underweight in the southwestern region (10).

The current reports examining the prevalence of overweight and obesity among adolescents in Saudi Arabia suggest similar rates of overweight and obesity among adolescents compared to Saudi Arabian adults. Interestingly, in a Saudi nationwide study that involved 12,463 adolescents, it was found that nearly 15% of the sample was underweight. The prevalence of underweight was higher among adolescent males (18%) compared to adolescent females (12.4%), suggesting a link between the higher prevalence of underweight, distorted body image, and abnormal eating behavior among adolescents (11).

Malnutrition can involve either a deficiency in or an excess of nutrient intake and can be associated with underweight, overweight, or obesity (12). Malnutrition during childhood is a public health issue in developing countries, leading to a double burden of malnutrition associated with both overweight and underweight (13). Several factors have been noted to be associated with malnutrition among adolescents, including low food diversification (14), skipping meals (15), maternal education (16), and socioeconomic status of the family (17, 18). In addition, adolescents’ self-perception can contribute to malnutrition, encouraging them to modify their weight.

Studies that assessed the frequency of body weight dissatisfaction among adolescents detected an alarmingly high prevalence. A recent systematic review aiming to determine the prevalence of body weight satisfaction among adolescents reported a high prevalence of dissatisfaction varying between 18 and 56% (19). Furthermore, a systematic review that assessed motivators of weight loss among adolescents concluded that the perception of having better health, cosmetic reasons, self-esteem improvement, and avoidance of bullying were primary motivators for weight loss among adolescents (20). A study that involved a sample of 3,249 adolescents from Poland indicated that underweight female adolescents are more likely to be satisfied with their body weight when compared to females with normal weight (21). Similarly, an Italian study that assessed body image perception among students aged between 11 and 14 indicated that females were more likely to be dissatisfied with their body image, and monitoring of body image perception among adolescents needs to address the prevention of nutritional disorders (22). This implies a tendency among some adolescents to adopt an underweight lifestyle behavior, which may have future health implications.

Perception about body weight has been indicated to have an influence on physical activity, and dieting behavior among adolescents. In a study that involved a sample of 180 adolescents from Mauritius, where 78 adolescents tried to lose weight (43.3%). Among those who tried to lose weight, 88.5% perceived themselves as overweight, while only 19.2% of them were actually overweight. The perception of being overweight was associated with higher attempts to reduce fat intake, exercise, reduce sugar intake and to increase intake of fruits and vegetables (23). Nonetheless, another larger scale study that involved a sample of 19,332 students aged between nine and 12 years from Canada reported different findings, where boys who perceive themselves to be overweight were less likely to meet the recommended level of physical activity compared to those who perceive themselves to have a normal weight, and girls who perceive themselves to be overweight were less likely to consume fruits and vegetables in comparison to girls who perceive themselves to have a normal weight, suggesting that strategies aiming to increase awareness about weight status among adolescents in Canada might not be sufficient for obesity prevention among adolescents (24). In a similar American study that involved a sample of 12,016 students aged between <14 and 18, it was also reported that adolescents who misperceive their weight are likely to participate in unhealthy dietary behaviors and engage in less level of physical activity (25). However, given the observed variation in the influence of body weight perception on lifestyle choices of adolescents, it is possible to argue that the influence of perception about body weight might have different levels of effect on dieting behavior and physical activity levels depending on the motivation to adopt weight modification measures.

Perception about body weight can contribute to body weight modification attempts among adolescents. Some adolescents may adopt a lifestyle behavior aiming to modify their body weight despite having a normal weight. Furthermore, some adolescents might utilize methods to alter their body weight without seeking a consultation from a healthcare professional. There is limited evidence about the experience of adolescents in Saudi Arabia concerning their body weight modification practices. This study assesses adolescents’ body weight satisfaction, perception, and body weight modification experiences in southwestern Saudi Arabia, including attempts to lose or gain weight.

## Methodology

### Study context

This study’s cross-sectional design targeted adolescents registered in intermediate and secondary schools in the Jazan region, southwest of Saudi Arabia. The recruitment occurred online after securing parental informed consent. An accompanying information sheet was provided for parents and explained the study objectives and measurement process. Data collection was performed while maintaining confidentiality of the parents and their children and did not involve any collection of identification data. Parents and their children had the right to refuse participation and to withdraw at any stage of the study’s recruitment. Ethical approval to conduct the study was granted via the Standing Committee for Scientific Research of Jazan University (approval number REC-44/06/446, dated December 2022). Data collection was performed during February and March 2023.

### Data collection tool

A questionnaire was constructed to measure the adolescents’ demographics and their experience concerning their weight modification attempts. Items measuring the demographic and clinical characteristics were adopted from the literature that assessed determinants associated with body weight among adolescents in Saudi Arabia such as gender, family size, living with the parents and diagnosis with a chronic disease (6, 11). Furthermore, they were asked about their body weight and height to enable an estimation of their BMI percentile according to age. In addition, they were asked about their body weight satisfaction, their perception of their body weight, and their intention to modify their body weight. They were further asked whether they attempted to modify their weight during the past 3 years, their body weight goals, whether they could modify it, and if they could maintain the modification they made. Finally, the adolescents were asked about their method of modifying their weight and receipt of support to modify their body weight from family, friends, school, and the community. The questionnaire was piloted on a sample of 10 male and 10 female adolescents to test the clarity of the questions and the time needed to complete the questionnaire.

### Data collection process

The questionnaire was transformed into an online form via Google Forms. A web link was generated to enable access to the study’s questionnaire. Identification and approaching were completed via distributing the questionnaire in the relevant social media to target parents in the Jazan region. Parents who consented to their children’s participation were given access to the questionnaire, enabling their adolescents to complete it. Adolescents registered at intermediate and secondary schools in the Jazan region who tried to modify their weight during the past 3 years were included in the analysis. Adolescents older than 19 and those who had not attempted to modify their body weight during the past 3 years were excluded.

Convenient, non-random sampling was utilized to reach the required sample for the study. Parents were asked to share the generated web link with other parents in the region to facilitate the data collection process and achieve the sample size. Only one study was identified to assess weight loss attempts among adolescents in the Saudi community. However, their assessment was only based on weight loss attempts among those who are already classified as underweight or normal weight and did not provide a prevalence concerning those who are overweight and obese (11). Therefore, a prevalence of weight loss attempts among adolescents with variable weight categories from the UK was used for a sample size estimation for the current study. Ahmed et al. found that weight loss attempts among adolescents from the UK reached 26.5% during 2015–2016 (26). Using the StatCal function of Epi Info, a sample of 299 adolescents was estimated by assuming an expected frequency of weight modification attempts among the adolescents of 26.5%, a 95% confidence interval, and a 5% margin of error.

### Data analysis

Data was analyzed using the Statistical Package for the Social Sciences, version 25. Binary and categorical variables were summarized by frequencies and proportions. Assessment of the adolescents’ body weight, including current and targeted body weight, according to height and age, was performed using the BMI Percentile Calculator for Child and Teen from the Centers for Disease Control and Prevention. The chi-square test was used to assess the association between the ability to modify weight and maintain the modification according to the ability to set an appropriate weight target based on the age of the adolescents, method of weight modification, and receipt of support to modify weight. The tests of the associations were based on the assumption of detecting a difference concerning the success to modify body weight or to maintain the modification according to the appropriateness of the weight target, method of weight modification, and receipt of support to modify weight. A *p* value of 0.05 was utilized to indicate statistically significant tests.

## Results

### Participants’ characteristics

A total of 285 adolescents from the region were recruited in the current investigation. The demographic characteristics of the sample are displayed in Table 1. More than half of the sample were female (58%) and living in urban areas (56%). Most of the respondents were secondary school students (73%) living with both of their parents (83%). More than half indicated that their families comprised four or fewer siblings (61%). When the adolescents were asked whether they suffered from a diagnosed chronic condition, the most frequently reported condition was obesity (6.3%), followed by asthma (5.3%). Finally, nearly half of the adolescents noted a parental history of a chronic disease (51%).

**Table 1:** Demographic characteristics and history of chronic diseases among 285 intermediate and secondary school students from Jazan, Saudi Arabia.

Gender	
Male	119 [41.8%]
Female	166 [58.2%]
Residence area	
Rural	124 [43.5%]
Urban	161 [56.5%]
Education level	
Intermediate	77 [27%]
Secondary	208 [73%]
Living with the parents*	
With both parents	237 [83.2%]
With the father only	11 [3.9%]
With the mother only	32 [11.2%]
Number of siblings in the family	
4 or less	174 [61.1%]
More than 4	111 [38.9%]
Diagnosis with a chronic disease	
Frequent dental illness	13 [4.6%]
Mental illness	6 [2.1%]
Obesity	18 [6.3%]
Asthma	15 [5.3%]
Sickle cell disease	9 [3.2%]
Diabetes	4 [1.4%]
Parental history of chronic disease	147 [51.6%]

*5 missing cases.

BMI category, body weight satisfaction and perception, and intentions of body weight modifications are displayed in Table 2. Among the recruited sample, 52% had a normal body weight, 12.6% were underweight, and 32.3% were overweight or obese. Most of the recruited sample were unsatisfied with their body weight (63%). Although the estimated BMI percentile for age and height indicated that 52% had a normal BMI, only 35% perceived their body weight as normal. When the adolescents were asked about their intentions concerning body weight modification, nearly half indicated that they intended to lose weight (52%), while 17% reported their intention to gain weight, and 21% of the sample indicated that they needed to make an effort to maintain their body weight.

**Table 2:** BMI category, body weight satisfaction and perception, intention, and practice of body weight modification among 285 intermediate and secondary school students from Jazan, Saudi Arabia.

Statement	Frequency [proportion]
BMI category	
Underweight	36 [12.6%]
Normal	149 [52.3%]
Overweight or obese	92 [32.3%]
Body weight satisfaction	
Yes	105 [36.8%]
No	180 [63.2%]
Perception of current body weight	
My body weight is low	47 [16.5%]
My body weight is normal	101 [35.4%]
My body weight is large	137 [48.1%]
Intention concerning body weight modification	
To do nothing concerning body weight	30 [10.5%]
To increase body weight	48 [16.8%]
To maintain the current body weight	59 [20.7%]
To reduce the body weight	148 [51.9%]

Table 3 describes the respondents’ experience with weight modification. When the adolescents were asked about their body weight modification attempts during the past 3 years, most indicated that they attempted to lose weight (76.5%). Among the 218 adolescents who attempted to lose weight, 164 (75%) reported that they had. Additionally, among those who lost weight, less than half of the sample (80 adolescents) could maintain the weight loss they achieved. Finally, when the adolescents who attempted to lose weight were asked about their desired weight loss target, 52 selected abnormal body weight targets (including underweight and overweight).

**Table 3:** Body weight modification experience among 285 intermediate and secondary school students from Jazan, Saudi Arabia.

Statement	Frequency [proportion]
Body weight modification attempts during the past 3 years	
To reduce body weight	218 [76.5%]
To increase body weight	67 [23.5%]
Students who were able to lose weight (proportion among those who tried to lose weight)	164 [75%]
Students who were able to gain weight (proportion among those who tried to gain weight)	51 [76%]
Appropriateness of the desired weight loss target*	
Underweight target	26 [13.6%]
Normal weight target	139 [72.8%]
Overweight weight target	26 [13.6%]
Appropriateness of the desired weight gain target**	
Underweight target	5 [13.5%]
Normal weight target	29 [78.4%]
Overweight weight target	3 [8.1%]
Students who were able to maintain body weight after weight loss (proportion among students to lost weight)	80 [48.7%]
Students who were able to maintain body weight after weight gain (proportion among students to gained weight)	35 [68.6%]

*27 missing cases, valid percent.

**30 missing cases, valid percent.

Among the respondent sample, 67 (23.5%) attempted to gain weight, of whom 51 (76%) were able to gain weight. Among the students who were able to gain weight, only 35 (69%) could maintain the weight they gained. When the adolescents aiming to gain weight were asked about their desired body weight gain targets, eight selected abnormal body weight targets. Collectively, among the adolescents who succeeded in modifying their body weight, only 53% (115) could maintain the modification.

Table 4 displays body weight modification methods. The most frequently selected body weight modification method was dieting (83%), followed by exercise (69%). Only 40 adolescents (14%) reported consulting a physician regarding their body weight modification attempts. When the adolescents were asked about sources of support for body weight modification, the most frequently reported source of weight modification was the family (51%), followed by the availability of community facilities supporting body weight modification (49%), while the lowest frequency of support was reported concerning the schools’ contribution (29%).

**Table 4:** Body weight modification methods and receipt of support among 285 intermediate and secondary school students from Jazan, Saudi Arabia who attempted to modify their body weight.

Statement	Frequency [proportion]
Body weight modification method	
Diet	236 [82.8%]
Exercise	68.8 [68.8%]
Pills	25 [8.8%]
Consulting a physician	40 [14%]
Herbal medications	32 [11.2]
Receipt of support for body weight modification	
Family	146 [51.2%]
Friends	121 [42.5%]
School	82 [28.8%]
Community members	108 [37.9%]
Facilities	139 [48.8%]

Table 5 displays associations between the ability to modify body weight and maintain the modification according to the appropriateness of the weight modification target, method of the modification, and receipt of support. Higher frequencies of adolescents selected an appropriate weight modification target and utilized dieting methods were able to modify their body weight. However, the associations were not statistically significant (*p* > 0.05). Nonetheless, among the adolescents who were able to maintain their body weight modification, a statistically significant difference was detected, indicating higher frequency among adolescents who selected dieting methods to modify weight compared to those who did not utilize dieting (*p* < 0.005).

**Table 5:** Association between the ability to modify body weight, ability to maintain modified body weight according to modification target, method of weight modification, and receipt of support to modify weight among 215 intermediate and secondary school students from Jazan, Saudi Arabia.

	Ability to modify weight frequencies [proportions]			Ability to maintain modified weight frequencies [proportions]		
	Yes	No	*p* value*	Yes	No	*p* value *
Targeted body weight appropriateness^**^ 0.35						0.14
Appropriate target	143 [74.9%]	25 [67.6%]		69 [70.4%]	74 [79.6%]	
Inappropriate target	48 [25.1%]	12 [32.4%]		29 [29.6%]	19 [20.4%]	
Method of body weight modification						
Diet			0.14			0.005
Yes	182 [84.7%]	54 [77.1%]		90 [78.3%]	92 [92%]	
No	33 [15.3%]	16 [22.9%]		25 [21.7%]	8 [8%]	
Pills			0.67			0.09
Yes	18 [8.4%]	7 [10%]		13 [11.3%]	5 [5%]	
No	197 [91.6%]	63 [90%]		102 [88.7%]	95 [95%]	
Exercise			0.35			0.33
Yes	151 [40.2%]	45 [64.3%]		84 [73%]	67 [67%]	
No	64 [29.8%]	25 [35.7%]		31 [27%]	33 [33%]	
Consulting a physician			0.74			0.58
Yes	31 [14.4%]	9 [12.9%]		18 [15.7%]	13 [13%]	
No	184 [85.6%]	61 [87.1%]		97 [84.3%]	87 [87%]	
Herbal medications			0.41			0.96
Yes	26 [12.1%]	6 [8.6%]		14 [12.2%]	12 [12%]	
No	189 [87.9%]	64 [91.4%]		101 [87.8%]	88 [88%]	
Receipt of support concerning body weight modification						
Family			0.43			0.69
Yes	113 [52.6%]	33 [47.1%]		59 [51.3%]	54 [54%]	
No	102 [47.4%]	37 [52.9%]		56 [48.7%]	46 [46%]	
Friends			0.3			0.82
Yes	95 [44.2%]	26 [37.1%]		50 [43.5%]	45 [44.2%]	
No	120 [55.8%]	44 [62.9%]		65 [56.6%]	55 [55%]	
School			0.38			0.43
Yes	59 [27.4%]	23 [32.9%]		29 [25.2%]	30 [30%]	
No	156 [72.6%]	47 [67.1%]		86 [74.8%]	70 [70%]	
Community members			0.89			0.93
Yes	81 [37.7%]	27 [38.6%]		40 [34.8%]	41 [41%]	
No	134 [62.3%]	43 [61.4%]		75 [65.2%]	59 [59%]	
Community facilities			0.25			0.93
Yes	109 [50.7%]	30 [42.9%]		58 [50.4%]	51 [51%]	
No	106 [49.3%]	40 [57.1%]		57 [49.6%]	49 [49%]	

*Chi-square tests, 5 missing cases. **24 missing cases.

## Discussion

This study aimed to evaluate body weight modification experience among Saudi Arabian adolescents. The majority of respondents were not satisfied with their body weight despite some having a normal body weight. Additionally, perception of body weight was associated with the selection of abnormal body weight modification targets, including underweight and overweight targets, suggesting the presence of distorted body image among this sample of adolescents. Nearly three-quarters of the sample were able to modify their body weight. However, fewer were able to maintain the modification. Upon assessing factors associated with the ability to modify weight or maintain the modification, only adolescents who indicated the modification of dieting behavior were able to maintain the modification compared to other weight modification practices (*p* < 0.05).

The current study’s findings can be compared to similar national or international investigations. A study conducted by Abalkhail et al. (27), which recruited a sample of 2,860 students from Jeddah schools in Western Saudi Arabia, concluded that nearly 26% of the sample had a distorted body weight perception; females were more likely to perceive themselves as overweight than males and were more likely to try to lose weight. This aligns with our findings, which indicated that some adolescents might have distorted perceptions about their body weight, leading them to adopt weight modification practices. Furthermore, Abalkhail et al. noted that some females adopted extreme lifestyle modifications, including fasting for 24 h, suggesting the importance of initiating nutritional education intervention programs in schools to promote awareness about ideal body image and prevention of harmful weight control measures (27).

Over half of the students recruited for our study exhibited normal body weight according to age and height. Nonetheless, nearly two-thirds of them perceived their body weight to be abnormal. Similar international studies’ findings indicate a potentially harmful influence of distorted body image on adolescents’ lifestyle choices. A systematic review of 30 articles that evaluated adolescent body image and dietary habits concluded that some adolescents tend to either underestimate or overestimate their body weight. The wrong estimation of the ideal body weight was associated with unhealthy dietary habits (28). Similarly, a large-scale longitudinal European study targeting more than 600,000 adolescents aged 11, 13, and 15 years concluded that higher BMIs and overestimating body weight were associated with a higher prevalence of weight reduction behaviors (29).

Our findings determined that most respondents were unsatisfied with their body weight. Although our study did not investigate factors associated with that dissatisfaction, the current literature assessing body image perception among adolescents demonstrates that several factors can be related to the dissatisfaction and development of negative images about weight, such as weight-related bullying and transitions associated with puberty and body appearance (30). Additionally, a study that assessed variation in trends of body weight perception among adolescents during five intervals between 2002 and 2018 reported that underestimation of weight status increased while overestimation of weight status decreased among adolescents (31). Furthermore, a UK study that assessed weight loss attempts among 34,235 children aged between eight and 17 concluded that it increased in 2015–2016 compared to 1997–1998; this rise was not justified by an increase in the prevalence of higher BMI and signified that some healthy children attempted to lose weight (26). Although our study has a cross-sectional nature and does not compare changes in trends, our results show that the proportion of adolescents who selected underweight targets for their body modification attempts was similar to those who chose overweight targets. The majority were able to set normal targets for their body modification attempts.

The presence of discrepancy between normal body weight according to the age and height and how the adolescents perceive their body weight can be explained by several factors. Perception of body weight can be influenced by weight stigma and body shaming. The current evidence indicates a growing influence of social media in shaping beliefs and the higher risk of exposure to weight stigmatization materials leading to problematic perception about weight (32). In a study that involved a sample of 11,384 adolescents from Saudi Arabia indicated that the duration of smart phone use was associated with distorted body image and adoption of weight loss behaviors (33). Similarly, in a study that assessed the exposure of 452 adolescents aged between 11 and 17 from the US to weight stigmatization from peers, parents and social media platforms during the COVID-19 pandemic reported that more than 74% of the adolescents received teasing comments about their weight and more than half of the adolescents were exposed to a minimum of one form of weight stigmatization contents on social media (34). The influence of weight victimization among adolescents has been indicated to negatively affect physical activity where some adolescents may avoid engaging in physical activities due to being teased and bullied by peers leading to enhanced feeling of humiliation and insecurity about appearances (35).

The findings of the current study indicated that the majority are living with their parents. It is possible to argue that living with the parents may have an impact on body weight satisfaction among the adolescents. However, influence of living with the parents and influence of living in urban or rural areas on body weight perception among the adolescents is currently lacking in a Saudi Arabian context. Nonetheless, in a study that assessed body weight satisfaction among adolescents in 24 American and European countries concluded that body weight satisfaction among the adolescents can be influenced by parents communications where difficulty of talking to father was associated with body weight dissatisfaction among the adolescents (36). Furthermore, the current evidence suggests that body weight among the adolescents can be influenced by weight status of the parents where increased parents weight is a risk factor for raised body weight among their children (37). Additionally, the influence of living with the parents was indicated to be associated with raised weight among the adolescents where adolescents living in one-parent families are at higher risk of raised body weight (38).

The findings of the current study indicated that more than half of the students were living in urban areas. It is possible to argue that living in urban or rural areas might be associated variations in the body weight satisfaction among the adolescents due to possible socio-cultural variations between Saudi urban and rural areas. However, assessment of influence of the living area on body weight satisfaction in a Saudi Arabian context is currently lacking. Nonetheless, the current literature indicates the presence of conflicting findings concerning factors influencing adolescents body weight in urban or rural areas. For example, in a study that involved 60,040 students from Korea concluded that raised body weight among urban adolescents is influenced by their access to fast food in comparison to those living in rural areas (39). However, in a US study that involved a sample of 23,199 participants aged between 10 and 17 years, it was concluded that prevalence of raised body weight was higher among adolescents in rural areas, and is likely to be attributed to the limited access to weight control programs in rural areas in comparison to urban areas (40). In another study that compared body weight among Malawi adolescents from urban and rural areas concluded that raised body weight was higher among adolescents from urban areas and the body weight perception was not statistically different between adolescents living in urban and rural areas (41). These conflicting findings suggests the presence of variation of impact socio-cultural levels associated with area of living on adolescents body weight.

Our research demonstrated that children who adopted dieting practices were more likely to have reported maintenance of the achieved body weight modification. This may suggest that the adolescents who relied on dieting behavior modification practices were more successful than those who used physical activity practices for body weight maintenance. Our findings are supported by a nationwide study involving a sample of 12,500 adolescents from different regions in Saudi Arabia that concluded that 44% do not engage in physical activity practices, suggesting an urgent need to enhance physical activity promotion among the age group (42). However, studies that assessed maintenance of modified body weight among adolescents in a Saudi context are currently lacking. Nonetheless, findings from international contexts indicated the presence of factors that can affect maintenance of the modified body weight among adolescents. A US study involved a sample of 1,902 adolescents and followed them up for 10 years concluded that adolescents who used unhealthy weight control behaviors, such as use of laxatives, diet pills, vomiting, and improper dieting, were more likely to regain weight over time (43). In another US study that involved a sample of 49 adolescents to assess their weight loss attempts, it was concluded that parents involvement and age of the adolescents were associated with the success to maintain weight loss (<0.05) (44). In a review that assessed weight loss maintenance among adolescents, it was stressed that more effort should be made to teach the adolescents about weight loss maintenance skills (45). However, evidence concerning impact of societal pressure, unrealistic body image, and influence on social media on weight modification maintenance among the adolescents is currently limited and is an area for future research.

The current investigation’s results noted that a minority of adolescents consulted a physician concerning their body weight modification attempts. This may partially explain the presence of a distorted body weight image among adolescents and the need to enhance the contribution of Saudi Arabian healthcare establishments to promote healthy weight practices. This finding is supported by the findings of similar US study that involved a sample of 2,793 adolescents and concluded that adolescents who have good self-esteem and high body satisfaction are more likely to practice healthy weight control measures in comparison to those who are having lower self-esteem and low body satisfaction suggesting the need to provide psychological support for adolescents to enhance the adoption of healthy weight control practices (46). Additionally, in another US study that involved a sample of 9,943 adolescents to evaluate weight control behaviors, it was reported that adolescents whom are overweight are more likely to adopt unhealthy weight control practices compared to those with normal weight indicating that clinicians should be aware that adolescents with overweight can adopt unhealthy weight management practices where this awareness should be reflected on prevision of screening services to identify adolescents at risk of practicing unhealthy weight control measures and to facilitate provision of healthy lifestyle interventions (47).

Adolescents’ limited utilization of healthcare services in the region might be partially explained by their limited access to healthcare services. This notion is supported by the findings of a study by Najjar et al.,(48) which concluded that 25% of adolescents in Saudi Arabia reported difficulty accessing healthcare; females, younger adolescents, and adolescents in families with a larger number of siblings were more likely to report access of healthcare difficulty. In addition to difficulty of access to healthcare, quality of healthcare service provided to adolescents to for weight control practices can be influenced by how healthcare service is delivered. In a qualitative focus group study that involved a sample of 16 multidisciplinary Canadian physicians to explore implementation of clinical practice guidelines for weight management services for adolescents with obesity, it was concluded that weight management services provided to the adolescents should be based on realistic expectations, should assess and address personal barriers of each adolescent on individual basis, and should address social pressure caused by peers, family, and the media and to understand how this pressure can influence perception of the adolescents about their weight (49).

## Strengths and limitations

This assessment has several areas of strengths and weaknesses. Its main strength is its ability to reach a sample of adolescents with variable intentions and experiences concerning their body weight modification efforts. Additionally, this study was able to reach a sample of adolescents with varying demographic characteristics; this would have been difficult to obtain if the recruitment had been performed in clinical settings due to the low utilization of healthcare services for weight modification. The main weaknesses of this research are related to the possibility of selection bias, where some families would have limited access to the internet and thus a smaller chance to participate in the study. Therefore, the possibility of selection bias limits the generalizability of the current study. Nonetheless, it is possible to argue that identification of similar local and international findings supported the findings of the current study and enabled performing several comparisons with reasonable generalizability while considering methodological differences. Additionally, information bias may have occurred when assessing body weight parameters due to the effect of social desirability and reporting bias. Nonetheless, we argue that our investigation has an acceptable external validity as the body weight dissatisfaction and misperception of body weight status identified in the current study are similar to other local and international literature.

## Conclusion

The evidence generated from the current study is consistent with similar studies conducted in local and international contexts, suggesting the presence of high levels of body weight dissatisfaction and misperceptions about body weight among adolescents. The misperception can be associated with adopting body weight modification behaviors that might not have been necessary and the limited ability to maintain the modification. This is augmented by the findings related to the limited contribution of healthcare professionals in adolescents’ body weight modification experiences. The results of the current study indicate a need for further research to identify factors associated with body weight misconception among adolescents in the region. Additionally, further research is needed to understand the influence of societal pressure on development of unrealistic body image, and the influence of social media on body weight modification experiences among Saudi adolescents. Additionally, future research should investigate barriers of seeking healthcare services among the adolescents and the importance of development of suitable interventions for weight management among the adolescents in the Saudi community. Finally, the findings highlight the importance of collaboration between families, schools, and healthcare services to improve adolescent body image and prevent the adoption of unhealthy body weight modification practices.

## Data availability statement

The raw data supporting the conclusions of this article will be made available by the authors, without undue reservation.

## Ethics statement

The studies involving humans were approved by Standing Committee for Scientific Research of Jazan University (approval number REC-44/06/446, dated December 2022). The studies were conducted in accordance with the local legislation and institutional requirements. Written informed consent for participation in this study was provided by the participants’ legal guardians/next of kin.

## Author contributions

IG: Conceptualization, Data curation, Formal analysis, Investigation, Methodology, Project administration, Validation, Writing – original draft, Writing – review & editing.
